# Programmed Death Ligand 1 (PD-L1) Expression in Lymphomas: State of the Art

**DOI:** 10.3390/ijms25126447

**Published:** 2024-06-11

**Authors:** Magda Zanelli, Valentina Fragliasso, Paola Parente, Alessandra Bisagni, Francesca Sanguedolce, Maurizio Zizzo, Giuseppe Broggi, Stefano Ricci, Andrea Palicelli, Moira Foroni, Fabrizio Gozzi, Pietro Gentile, Andrea Morini, Nektarios Koufopoulos, Rosario Caltabiano, Luca Cimino, Massimiliano Fabozzi, Alberto Cavazza, Antonino Neri, Stefano Ascani

**Affiliations:** 1 Pathology Unit, Azienda USL-IRCCS di Reggio Emilia, 42123 Reggio Emilia, Italy; alessandra.bisagni@ausl.re.it (A.B.); stefano.ricci@ausl.re.it (S.R.); andrea.palicelli@ausl.re.it (A.P.); moira.foroni@ausl.re.it (M.F.); alberto.cavazza@ausl.re.it (A.C.); 2 Laboratory of Translational Research, Azienda USL-IRCCS di Reggio Emilia, 42123 Reggio Emilia, Italy; valentina.fragliasso@ausl.re.it; 3 Pathology Unit, Fondazione IRCCS Casa Sollievo della Sofferenza, 71013 San Giovanni Rotondo, Italy; p.parente@operapadrepio.it; 4 Pathology Unit, Policlinico Riuniti, University of Foggia, 71122 Foggia, Italy; francesca.sanguedolce@unifg.it; 5 Surgical Oncology Unit, Azienda USL-IRCCS di Reggio Emilia, 42123 Reggio Emilia, Italy; maurizio.zizzo@ausl.re.it (M.Z.); andrea.morini@ausl.re.it (A.M.); massimiliano.fabozzi@ausl.re.it (M.F.); 6 Department of Medical and Surgical Sciences and Advanced Technologies “G.F. Ingrassia” Anatomic Pathology, University of Catania, 95123 Catania, Italy; giuseppe.broggi@phd.unict.it (G.B.); rosario.caltabiano@unict.it (R.C.); 7 Ocular Immunology Unit, Azienda USL-IRCCS di Reggio Emilia, 42123 Reggio Emilia, Italy; fabrizio.gozzi@ausl.re.it (F.G.); pietro.gentile@ausl.re.it (P.G.); luca.cimino@ausl.re.it (L.C.); 8 Second Department of Pathology, Medical School, National and Kapodistrian University of Athens, Attikon University Hospital, 15772 Athens, Greece; koufonektar@yahoo.com; 9 Department of Surgery, Medicine, Dentistry and Morphological Sciences, University of Modena and Reggio Emilia, 41125 Modena, Italy; 10 Scientific Directorate, Azienda USL-IRCCS di Reggio Emilia, 42123 Reggio Emilia, Italy; antonino.neri@ausl.re.it; 11 Pathology Unit, Azienda Ospedaliera Santa Maria di Terni, University of Perugia, 05100 Terni, Italy; s.ascani@aospterni.it

**Keywords:** PD-1/PD-L1, lymphoma, cHL, DLBCL, PMLBCL, ALCL, PTCL, Epstein–Barr virus

## Abstract

The interaction of programmed death-1 (PD-1) on T lymphocytes with its ligands Programmed Death Ligand 1 (PD-L1) and Programmed Death Ligand 2 (PD-L2) on tumor cells and/or tumor-associated macrophages results in inhibitory signals to the T-cell receptor pathway, consequently causing tumor immune escape. PD-L1/PD-L2 are currently used as predictive tissue biomarkers in clinical practice. Virtually PD-L1 levels expressed by tumor cells are associated with a good response to immune checkpoint blockade therapies targeting the PD-1/PD-L1 axis. These therapies restore T-cell antitumor immune response by releasing T-lymphocytes from the inhibitory effects of tumor cells. Immune checkpoint therapies have completely changed the management of patients with solid cancers. This therapeutic strategy is less used in hematological malignancies, although good results have been achieved in some settings, such as refractory/relapsed classic Hodgkin lymphoma and primary mediastinal large B-cell lymphoma. Variable results have been obtained in diffuse large B-cell lymphoma and T-cell lymphomas. Immunohistochemistry represents the main technique for assessing PD-L1 expression on tumor cells. This review aims to describe the current knowledge of PD-L1 expression in various types of lymphomas, focusing on the principal mechanisms underlying PD-L1 overexpression, its prognostic significance and practical issues concerning the evaluation of PD-L1 immunohistochemical results in lymphomas.

## 1. Introduction

The programmed death 1 *(PD1)/PD ligand (PD-L)* pathway represents a relevant checkpoint regulating T-cell-mediated immune responses. It consists of the transmembrane protein PD-1/CD279 and the two ligands PD-L1, also called CD274 or B7-H, and PD-L2, also called CD273 or B7-DC [1]. PD-1 is expressed on the surface of activated T-lymphocytes. The interaction between PD-1 on T-cells and the two ligands results in a reversible inhibition of T-cell immune activity [2]. The balance of the immune system is based on the coordination between stimulatory and inhibitory signals. In healthy people, PD-1 and its ligands are crucial for preserving self-tolerance and avoiding autoimmunity. Antigen-presenting cells such as macrophages and dendritic cells normally express PD-L1.

To develop within an immunocompetent host, the neoplasms need to employ several mechanisms to escape the immune system defenses. One of the most frequent strategies involves the *PD-1/PD-L1/PD-L2* axis.

Several malignant neoplasms, including solid and hematopoietic tumors, can express PD-L1 on the cell surface [3]. PD-L1 expressed on neoplastic cells as well as on adjacent macrophages of microenvironment interacts with PD-1 on the T-cell surface. This interaction results in inhibition of T-cell-mediated immune response (so-called T-cell functional exhaustion) and leads tumor cells to escape from antitumor immune surveillance [4,5,6,7,8,9,10,11]. The current immunotherapy is based on the use of antibodies blocking the PD-1/PD-L1 pathway, so-called anti-PD-1/PD-L1 immune checkpoint inhibitors (ICIs). The blockade of the *PD-1/PD-L1* axis by ICIs releases T-cells from the inhibitory effect of tumor cells, restoring the T-cell antitumor function and resulting in tumor cell elimination [8]. Over the years, ICIs have revolutionized the treatment of cancers, changing the management of several neoplasms and achieving optimal and durable responses, in particular in solid tumors, including for instance lung cancer, breast cancer, and melanoma [12].

In hematological neoplasms, this therapeutic strategy is less used, although promising results have been obtained in some categories of lymphomas.

Many of the studies on PD-L1 expression in lymphomas have been performed on classic Hodgkin lymphoma (cHL) and B-cell lymphomas [13]. PD-L1 expression has been detected, for instance, in neoplastic cells of cHL, primary mediastinal large B-cell lymphoma (PMLBL) and EBV-positive diffuse large B-cell lymphoma (DLBCL) [13].

The *PD-1/PD-L1* pathway has been less evaluated in lymphomas of T-cell origin, with a limited number of studies in particular on PD-L1 expression in anaplastic large-cell lymphoma (ALCL), including cell lines and patient tissue specimens [14,15,16,17,18,19,20].

**Figure 1:**
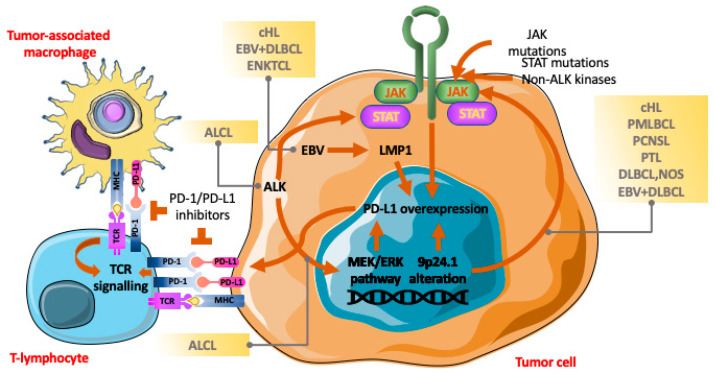
The PD-1/PD-L1 axis and interactions among tumor cells, tumor-associated macrophages and T-lymphocytes. In lymphomas, different mechanisms lead to PD-L1 overexpression in tumor cells (chromosome locus 9p24.1 alterations; activation of the *JAK/STAT* pathway; EBV infection and the *MEK/ERK* pathway). The interaction of PD-L1 on both tumor cells and tumor-associated macrophages with PD-1 on T-lymphocytes causes inhibition of T-cell-mediated immune response favoring tumor escape from the antitumor immune system. Treatment with immune checkpoint inhibitors releases T-lymphocytes, restoring the T-cell antitumor function.

Different mechanisms form the basis of PD-L1 expression in lymphomas (Figure 1).

The aim of our paper is to summarize the current knowledge of PD-L1 expression in lymphomas, particularly focusing on cHL, DLBCL and ALCL and the potential benefit of ICI therapy in this series of malignancies.

## 2. Principal Issues in Assessing PD-L1 Immunohistochemical Expression in Lymphomas

The expression of PD-L1 can be measured by immunohistochemistry (IHC) staining of formalin-fixed paraffin-embedded (FFPE) tissue sections. PD-L1 expression can be found on neoplastic cells as well as on peritumoral immune cells (ICs) in different neoplasms, including lymphomas [13]. The level of PD-L1 expression can be used as a potential predictor of the therapeutic efficacy of anti PD-1/PD-L1 agents [21]. There are currently more commercially available diagnostic kits for targeting PD-L1, and the different antibodies used may influence the results. The most representative antibody clones are clone 22C3 (Dako, Agilent Technologies), clone SP263 (Ventana) and clone SP142 (Ventana Roche) [22,23,24]. Clone 22C3 is a sensitive antibody, probably the most widely used currently [23,25], whereas SP142 is the first clinically validated antibody [22,24]. These Abs, commonly applied on carcinomas, have been approved by the United States Food and Drug Administration (FDA) for determining the probability of benefit from anti-PD-L1 drugs [22,23,24,25].

In carcinomas, PD-L1 expression is associated with a poor outcome because of PD-L1’s suppressor role in tumor immunity, regardless of the cut-off values and of the type of Abs used for immunohistochemistry [26,27].

In lymphomas, some issues in evaluating PD-L1 expression should be considered. First, no diagnostic test has been systematically assessed and no standard scoring system for interpreting PD-L1 expression is available in lymphomas. The cut-off values of PD-L1 expression vary among different studies [13,14].

Secondly, the interpretation of PD-L1 expression may be particularly difficult in lymphomas because many peritumoral ICs may express PD-L1.

PD-L1-positive ICs may be so close to tumor cells that it may be difficult to define whether neoplastic cells are PD-L1 positive or not. The use of double staining in IHC may aid in defining the percentage of PD-L1-positive elements, either tumor cells or non-tumor cells.

The prognostic significance of PD-L1 expression in lymphoma is variable according to the different types of lymphomas, and, for evaluating the prognostic significance of PD-1/PD-L1 expression, consistency in the therapeutic strategies of the patients included in the studies is crucial [28,29,30].

Although PD-L1 IHC is traditionally used for predicting the clinical response to ICI therapy, other methods for PD-L1 measurement in FFPE tumors such as next-generation sequencing (NGS) and polymerase chain reaction (PCR) should be mentioned. To the best of our knowledge, there is a paucity of information on assays other than IHC measuring PD-L1 levels on tissues. It is worth noting the study by Conroy et al., in which the authors tested next-generation RNA sequencing (RNA-seq) to determine PD-L1 mRNA expression levels in a large series of cancer patients [31]. Conroy et al. concluded that measurement of PD-L1 mRNA expression by RNA-seq is a robust method, comparable to routinely used ICH both analytically and clinically in predicting response to ICI therapy. However, PD-L1 measured by RNA-seq should be further evaluated in studies including different types of tumors [31].

## 3. cHL and PD1/PD-L1 Axis

cHL is a lymphoma of B-cell origin, usually characterized by a minority of tumor cells, both mononuclear Hodgkin cells and bi- or multinucleated Reed–Sternberg cells (HRS), dispersed within an overwhelming inflammatory microenvironment of small-sized lymphocytes, often of T-cell origin, plasma cells, histiocytes and eosinophils.

PD-1 is expressed in tumor-infiltrating lymphocytes (TILs) rather than in HRS cells, and PD-1 expression on TILs of cHL is higher than on lymphocytes from healthy people [17,32]. Increased PD-1-positive TILs are associated with poor prognosis in cHL [32,33].

Menter et al. evaluated the diagnostic and prognostic role of PD-L1 expression in a large cohort of both cHL and B-cell lymphomas [13].

PD-L1 has been found to be expressed in 70–87% of cHL on both HRS and peritumoral ICs, in particular tumor-associated macrophages (TAMs) [13,17,34,35]. PD-L1 staining is considered positive when a clear membranous staining with occasional dots is observed in at least 5% of tumor cells [13]. In the study by Menter et al., PD-L1 was found to be expressed on more than 5% of tumor cells in approximately 70% cHL, 54% nodular lymphocyte-predominant Hodgkin lymphoma (NLPHL), 34% PMLBL and 31% diffuse large B-cell lymphoma (DLBCL) [13].

Difficulties in the evaluation of PD-L1 staining may be caused by the fact that both HRS cells and reactive ICs can express PD-L1 generally in a membranous pattern in cHL.

Although cHL neoplastic cells are easily differentiated from ICs on the basis of size and unequivocal morphology, PD-L1-positive ICs may be so closely packed around neoplastic cells that it may be rather complicated to define whether HRS cells express PD-L1 [36].

PD-L1 expression on HRS cells and TAMs is associated with poor outcome in cHL patients [34,35,36].

In cHL, genetic alterations (polysomy, copy gain, amplification) on chromosome 9p24.1, where *PD-L1*, *PD-L2* and *JAK2* are located, are the principal mechanism upregulating PD-L1 and PD-L2 expression [32,33]. Alterations of 9p24.1 also upregulate *JAK2* expression with the activation of the *JAK/STAT* signaling pathway leading to a further increase in PD-L1 expression [34,35].

However, in cHL, other mechanisms may activate the *PD1-PD-L1* pathway. Epstein–Barr virus (EBV), positive in a subset of cHL, is another mechanism of PD-L1 upregulation [37,38,39]. EBV-encoded latent membrane protein (LMP)-1 promotes the activator protein-1 (AP-1) and *JAK/STAT* signaling pathway to activate the enhancer and promoter elements of PD-L1, respectively [38]. Besides EBV activation, *JAK2*-related PD-L1 activation is also obtained through other alterations at genetic level and microRNA interferences [13,40].

As already mentioned, in cHL, PD-L1/L2 is expressed by both tumor cells and TAMs, in particular by TAMs located near HRS cells. PD-L1-positive TAMs interact with PD-1-positive T lymphocytes, causing T-cell inhibition and therefore favoring the escape of neoplastic cells from antitumor immune surveillance [17,41]. An elevated number of TAMs in cHL is associated with inferior overall survival (OS) [14].

PD-L1 expression provided a rationale for ICI therapy in cHL. In their outstanding study, Ansell et al., demonstrated that anti-PD-1 antibodies such as pembrolizumab and nivolumab are effective in relapsed/refractory (R/R) cHL with high response rates [42]. These results led to FDA approval of nivolumab and pembrolizumab in the setting of R/R cHL [43,44]. Additionally, anti-PD-1 therapies represent a promising strategy as consolidation treatment after autologous stem cell transplantation (A-SCT) [45]; anti-PD-1 antibodies combined with other therapies such as anti-CD30 brentuximab vedotin have been reported to have a synergic effect [46].

## 4. DLBCL, NOS, Other Aggressive Peripheral B-Cell Lymphomas and PD-1/PD-L1 Axis

DLBCL is the most common non-Hodgkin lymphoma (NHL) subtype worldwide, accounting for 30–40% of NHL cases [47,48].

DLBCL, named large B-cell lymphoma (LBCL) in the recent WHO classification, is a broad and very heterogeneous category, including entities with distinct clinicopathological and biological characteristics. It may involve lymph nodes as well as extra-nodal sites [47,48,49]. The definition of diffuse large B-cell lymphoma not otherwise specified (DLBCL, NOS) is used for cases not belonging to any specific variant of DLBCL. Histologically, DLBCL, NOS is characterized by a diffuse proliferation of large cells (centroblasts or immunoblasts) partially or completely effacing the architecture of the involved tissue. Based on cell-of-origin (COO) classification, DLBCL is classified into these distinct prognostic subgroups: germinal center B-cell (GCB)-like subtype, activated B-cell (ABC)-like subtype and unclassified subtype [47,48]. GCB-DLBCL shows the gene signature of normal germinal center B cells, whereas ABC-DLBCL has the gene signature of post-germinal center B cells. The ABC subtype is an aggressive disease with a worse prognosis compared to the GCB subtype [47,48].

Whereas PD-L1 expression is low or absent in most indolent peripheral B-cell lymphomas, approximately one-third of DLBCL expresses PD-L1 [13,50,51,52].

PD-L1 is expressed by both lymphoma cells and macrophages in DLBCL [52]. The frequency of PD-L1 expression in DLBCL is variable depending on the different cut-off values applied (ranging from 5% to 30%), the type of anti-PD-L1 monoclonal Abs and the compartment analyzed (tumor cells or microenvironment cells) [50,51,52].

To precisely define the percentage of PD-L1-positive elements in either tumor cells or non-tumor cells, a study by Kiyasu et al. used PD-L1/PAX5 double staining in DLBCL and found 10.5% of lymphoma cells expressing PD-L1, using a cut-off value of 30% and 15.3% of adjacent TAMs expressing PD-L1, using a cut-off value of 20% [52].

Compared to cHL, the rate of PD-L1 expression in DLBCL is globally lower (31% in DLBCL versus 70% in cHL) and this feature may be related to the infrequent alterations of chromosome 9p24.1 in DLBCL [50]. The presence of cytogenetic alterations of 9p24.1, such as gains or amplifications, is more common in the ABC subtype in which, consequently, PD-L1 expression is higher than in GCB-DLBCL [17,52,53,54]. Approximately 30% of ABC-DLBCLs carry *MYD88* mutations, resulting in the activation of the *JAK/STAT* pathway and PD-L1 expression [50]. Immunomodulatory treatment could represent a good option in ABC-DLBCL, which is an aggressive entity, often resistant to conventional therapy [13]. In general, monotherapy with PD-1/PD-L1 inhibitors in R/R DLBCL is rather discouraging compared to R/R cHL, whereas a better strategy in R/R DLBCL could be the combination of ICIs with other therapies [14].

PD-L1 expression is much more common in EBV-positive DLBCL compared to the EBV-negative counterpart, being observed in approximately two-thirds of cases [30,38,46,48]. EBV-positive DLBCL, NOS was named EBV-positive DLBCL in the elderly in the 2008 WHO classification, being initially described in individuals older than 50 years [55,56,57]. It was subsequently renamed, being identified in younger individuals and even children [58,59]. Both nodal and extra-nodal sites may be involved. In younger patients, the disease more often affects nodal sites and has a better outcome than in older patients [58,59]. In elderly individuals, the disease is predominantly extra-nodal and patients often have a poor prognosis [55,56,60].

Genetic alterations of chromosome locus 9p24-1 and activation of the *JAK/STAT* pathway result in the higher level of PD-L1 expression in EBV-positive DLBCL (77%) compared to the EBV-negative form [37,58].

The prognostic significance of PD-L1 expression in DLBCL is rather controversial. In a large series of 1200 DLBCL cases, Kiyasu et al. found that PD-L1-positive patients had an inferior overall survival than PD-L1-negative ones [52].

Additionally, DLBCLs with PD-L1-positive tumor cells and a low number of PD-1+ TILs had poorer outcomes than DLBCLs with PD-L1-negative tumor cells and a high number of PD-1+ TILs [52].

The expression of PD-L1 on tumor cells and microenvironment ICs seems to have a divergent impact on prognosis. EBV-negative DLBCL with PD-L1 expression on the microenvironment cells exhibit better behavior compared to PD-L1-negative cases [52]. EBV-positive DLBCL with PD-L1 expression on tumor cells follows an aggressive course [61].

PD-L1 expression has been found to be a common feature of EBV-associated lymphoproliferative diseases, which may therefore benefit from ICI treatment [62].

Genetic alterations of chromosome 9p24.1 and upregulated expression of PD-L1 have been found in specific subtypes of DLBCL including PMBCL, primary diffuse large B-cell lymphoma of the central nervous system (PCNSL) and primary testicular large B-cell lymphoma (PTL) [50,63,64].

PMBCL is a separate entity in both WHO and ICC classifications, accounting for 2–4% of all NHLs [47,48]. It has distinct clinicopathological features when it occurs in young adults, predominantly females presenting with compressing symptoms caused by an anterior mediastinal mass; in particular, the predominant site of PMBCL must be the anterior mediastinum [47,48].

The disease has a distinct molecular signature overlapping with cHL. Similarly to cHL, in PMBCL, genetic alterations in 9p24.1 and 2p16.1 leading to the *JAK-STAT* and *NF-kB* pathways are recognized as disease hallmarks [63,65]. Immune evasion is characteristically achieved through different genetic mechanisms involving chromosome 9p24.1 leading to PD-L1 overexpression. PD-L1 is expressed in 36–100% of PMBCLs [13,17]. Copy number gains and amplifications of chromosome 9p24.1 are not the unique mechanisms causing PD-L1 overexpression, as, unlike in DLBCL and cHL, the 9p24.1/PD-L1 locus is rearranged in 20% of PMBCLs [17,66]. PD-L2 expression, uncommon in DLBCL, is a distinguishing feature of PMBCL, related to *PDCD1LG2* copy gain [67].

PCNSL and PTL are distinct subtypes of DLBCL involving immune-privileged extra-nodal sites. These diseases share common genetic signatures and are usually resistant to conventional therapies [64]. Copy number gains at chromosome 9p24.1 are found in approximately half of these neoplasms with consequent PD-L1 expression [64].

In conclusion, since the frequency of alterations of chromosome 9p24.1 and expression of PD-L1 in DLBCL is low, it is not currently recommended to treat unselected DLBCL with ICIs. Selective subtypes of DLBCL, such as PMBCL, PCNSL and PTL, which show frequent alterations at chromosome 9p24.1 and PD-L1 and/or PD-L2 expression, may benefit from PD-1 inhibitors. In particular, pembrolizumab has been approved by the FDA for patients with R/R PMBCL with excellent results [68].

## 5. Peripheral T-Cell Lymphomas (PTCLs) and PD-1/PD-L1 Axis

PTCLs comprise a very heterogeneous group of neoplasms originating from mature T/NK cells, accounting for about 10% of NHLs in western countries and being far more frequent in Asia (25% of NHLs) [41,42]. Of PTCLs, extra-nodal NK/T-cell lymphoma (ENKTL), closely linked to EBV, is the most frequent in East Asia [47,48], whereas other more frequent subtypes are peripheral T-cell lymphoma not otherwise specified (PTCL, NOS), angioimmunoblastic T-cell lymphoma (AITL) and ALCL, either ALK-positive or ALK-negative [47,53]. Excluding ALK-positive ALCL and early-stage ENKTL, PTCLs are aggressive and often incurable diseases, resistant to conventional chemotherapy; therefore, there is an urgent need of new effective therapies.

PD-1 is recognized as a diagnostically valid marker in AITL and in PTCL with a T-cell follicular helper (TFH) phenotype according to the current WHO and ICC classifications [47,48]. PD-1 expression has been found to be higher in AITL (61.6%) than PTCL, NOS (39.3%) and ALK-negative ALCL (13.3%), whereas PD-1 positivity is almost absent in ALK-positive ALCL [69].

It is quite clear that the expression of PD-L1 is very heterogenous among different subtypes of peripheral T-cell lymphomas [70,71,72,73,74,75,76].

Panjwani et al. assessed PD-L1 expression in 702 lymphoma cases and found PD-L1-positive neoplastic cells in 80% of ALK-positive and ALK-negative ALCLs without making a distinction between the two groups, 80% of AITLs, 39% of ENKTLs and 26% of PTCL, NOS cases [72]. In 2021, Shi et al. evaluated which types of PTCLs showed a PD-L1 rate =/> 50%, and by subtype, a PD-L1 rate =/> 50% was identified in 78.9% of ENKTLs, 71.4% of ALK+ ALCLs, 38.5% of ALK-negative ALCLs and 35.7% of PTCL, NOS cases [73]. Patients with PD-L1 expression =/> 50% found more benefit from anti-PD-1 therapy [73]. In PTCLs, data on the prognostic role of PD-L1 expression are inconsistent with contrasting results [74,75,76].

Studies on ENKTL have reported a variable expression of PD-L1 in both tumor cells and TAMs, possibly related to EBV infection, which is strictly associated with ENKTL [70,71]. Some studies demonstrated that ICIs (for instance, pembrolizumab and nivolumab) are effective in ENKTL patients with advanced disease [70,71].

However, in ENKTL, the association between PD-L1 expression and the effectiveness of PD-1/PD-L1 inhibitors remains discrepant, and there are ENKTL cases with high PD-L1 expression showing disease progression despite ICI treatment, indicating that other immune checkpoint pathways hamper antitumor immunity. Recently, He et al. found that, beyond PD-L1, another immune checkpoint of the B7 family, V-domain immunoglobulin suppressor of T-cell activation (VISTA) is aberrantly expressed in ENKTL [76]. In the study by He et al., VISTA expression was more prevalent than PD-L1 in ENKTL. High PD-L1 expression was associated with poor prognosis and cases with high expression of both PD-L1 and VISTA had the worst outcome. VISTA expression is likely responsible for the resistance to PD-1/PD-L1 therapy in some ENKTL cases and could represent a potential target for treatment either alone or in combination with PD-L1 immunotherapy [76].

Tumor-infiltrating ICs are components of the tumor microenvironment, and of these cells, the best characterized are TILs and TAMs. Many studies have demonstrated a high number of PD1+ TILs in lymphomas, as previously mentioned in cHL and DLBCL [33,37]. In PTCLs (either PTCL, NOS or AITL), Kim et al. found PD-1 expression in both tumor cells and ICs in 63.2% of cases [77], and several studies showed high PD-1 expression in tumor-infiltrating ICs in ENKTL; however, no significant correlation was found between PD1+ TILs and prognosis [75]. The clinical significance of PD-L1+ ICs in the lymphoma microenvironment has been less studied. In 2020, Kim et al. found that 76.3% of PTCL had a double positivity for both PD-1 and PD-L1 in neoplastic cells and ICs and that PD-L1 expression was associated with poor prognosis in AITL [77].

## 6. ALCL and PD-1/PD-L1 Axis

ALCL includes a group of CD30-positive NHLs of T/null phenotype sharing some morphological features, in particular the “hallmark cells” with classical horseshoe-shaped nuclei diffusely and strongly expressing CD30 [47,48]. The upcoming fifth edition of the WHO’s classification and the ICC recognize the following entities: systemic ALCL further sub-divided into ALK-positive ALCL and ALK-negative ALCL; primary cutaneous ALCL (PC-ALCL); and breast-implant-associated ALCL (BI-ALCL) [47,48]. Although these entities share some common morphological characteristics, the clinical manifestations and behavior are different [47,48,78,79,80,81].

Many different therapies are used to treat ALCL patients including anti-CD30 Brentuximab vedotin and ALK inhibitors [82]. Although ALK+ ALCL has a better outcome than ALK-negative ALCL, even ALK+ ALCL, more often arising in children and young adults, shows a high rate of relapse (25–35% of cases) with several recurrences during the disease course [18,83,84]. ALK-negative ALCL follows a more aggressive course with less than 50% patients alive after 5 years [83,84]. New therapeutic strategies are therefore needed to improve the outcome of ALCL. Recently, our group explored the role of HELLS, a multifunctional chromatin remodeling protein affecting genomic instability, in ALCL, providing a potential rationale for treatment design [85,86].

In ALCL, the expression of PD-L1 is variable, ranging from 50% to 80%, with a higher positivity rate in ALK+ ALCL [16,87,88].

In 2017, Atsaves et al. demonstrated the expression of PD-L1 in 50% of ALK+ ALCLs and in 67% of ALK-negative ALCLs [20], whereas, more recently, a large study by Shen et al. detected PD-L1 expression more often in ALK+ ALCL (76%) than in ALK-negative ALCL (42%), using a 5% cut-off value for PD-L1 IHC positivity [16].

The mechanisms responsible for PD-L1 expression in ALCL are different from those observed in cHL and DLBCL because genetic alterations of the chromosome 9p24.1/*PD-L1/PD-L1* loci are not commonly detected in ALCL [20]. The presence or not of *PD-L1* amplification in ALCL was initially a matter of debate; however, recently, PD-L1 expression in ALCL is considered unrelated to *PD-L1* amplification or rearrangement [20,69].

Two signaling pathways are involved in controlling PD-L1 expression in ALCL.

The central pathogenetic event of both ALK-positive and ALK-negative ALCL is represented by the constitutive activation of the *JAK/STAT* pathway [16,89,90]. In the study by Khoury et al., nuclear pSTAT3 expression was observed in 50% of ALK-negative ALCLs and 85% of ALK-positive ALCLs [89]. PD-L1 expression is likely regulated by *STAT3* as PD-L1 expression correlates with STAT3 expression in tumor cells [16,90].

Among ALK-negative ALCL cases, *DUSP-22* rearranged ALK-negative ALCL cases are frequently lacking pSTAT3 and PD-L1 expression [91].

Another signaling pathway regulating PD-L1 expression in ALK+ ALCL is the *MEK/ERK* signaling pathway [92]. In ALK-positive ALCL, the fusion gene *NPM:ALK* leads to PD-L1 expression through activation of both STAT3 and the signalosome containing GRB2/SOS1 which activates the *MEK-ERK* pathway [16,19,88,92].

Interestingly, Iwafuchi et al., by using PD-L1/ALK immunofluorescence double staining, evaluated PD-L1 expression on tumor cells and peritumoral ICs in children with ALK-positive ALCL [18]. The progression-free survival (PFS) did not differ between cases with high or low PD-L1 expression on tumor cells and on peritumoral ICs. The study by Iwafuchi et al. demonstrated that pediatric ALK-positive ALCLs with high PD-L1 expression on tumor cells combined with the presence of many peritumoral ICs were associated with a poor outcome [18]. Therefore, in ALK-positive ALCL, the tumor microenvironment is likely to be important in the outcome of the disease.

Recently, clinical trials evaluated the antitumoral efficacy of Pembrolizumab combined with other therapies in R/R T-cell lymphomas including ALCL, AITL and PTCL with a TFH phenotype and demonstrated an overall response rate (ORR) of 50% [93]. In these trials, cases with higher PD-L1 expression achieved complete remission [93]. Meanwhile, there is concern that PD-1 pathway blockade might accelerate the growth of T-cell lymphomas in rare cases [92]. An increase in T-cell lymphoma frequency (0.02%) secondary to ICIs used in the treatment of solid neoplasms has been reported [94].

BI-ALCL is a rare form of ALCL arising in the capsule of patients with textured-surface breast implants [47,48]. Rearrangements of *ALK*, *DUSP22* and *TP63* classically found in systemic ALCL are absent in BI-ALCL. Similarly to systemic ALCL, the *JAK/STAT* pathway is relevant in the development of BI-ALCL and the activation of this pathway causes pSTAT3 and PD-L1 expression in this rare variant of ALCL [95,96,97,98]. A recent report identified PD-L1 copy number alterations in 33% of BI-ALCL [99]. The disease is potentially targetable by ICIs, due to the high PD-L1 expression (approximately 56% of cases) of BI-ALCL.

A very limited number of reports have addressed the issue of PD-L1 expression on primary cutaneous T-cell lymphoma (CTCL), in particular on PC-ALCL and mycosis fungoides (MF) [100,101]. Kentekure et al. reported increased PD-L1 expression in tumor cells in MF showing disease progression and large cell transformation [100]. Recently Takahashi et al., identified four cases of PC-ALCL that developed secondary nodal involvement and interestingly found that PD-L1 expression in neoplastic cells greatly increased with tumor progression to nodal involvement [101,102]. PD-L1 expression was =/>50% of lymphoma cells in nodal disease, whereas it was rarely detectable or very low (=/<1%) when the disease was limited to the skin [101,102]. These findings support the concept that neoplastic PD-L1 expression in CTCL increases with lymphoma progression. In addition, Takahashi et al. noted that the nodal lesions of PC-ALCL had a tumor microenvironment rich in PD-L1-positive TAMs with scarce PD-1 expression on T-cells very similar to that observed in cHL [102]. Although further studies are required to understand the mechanisms underlying PD-L1 expression in PC-ALCL and other CTCLs, the findings of high PD-L1 expression in tumor cells and TAMs of PC-ALCL with nodal involvement may have therapeutic implications for the use of ICIs in this setting of patients.

## 7. Conclusions

Over the past few years, ICIs have become one of the most attractive areas in oncology and have completely changed the management of cancer patients. ICIs have been approved for the treatment of different solid tumors. In hematological tumors, ICIs are less used, although these agents have become a standard treatment in some subgroups of lymphomas such as R/R cHL and R/R PMBL, and promising results have been obtained in R/R EBV-positive DLBCL and R/R ALCL.

## Data Availability

Individual patient data from the original studies included in the present review are not available and data sharing at this level is not applicable for a review.
